# Association between Reproductive Factors and Type 2 Diabetes: A Cross-Sectional Study

**DOI:** 10.3390/ijerph19021019

**Published:** 2022-01-17

**Authors:** Yuting Yu, Jing Li, Yonggen Jiang, Maryam Zaid, Qi Zhao, Na Wang, Xing Liu, Yun Qiu, Junjie Zhu, Xin Tong, Shuheng Cui, Yiling Wu, Jianguo Yu, Genming Zhao

**Affiliations:** 1 Key Laboratory of Public Health Safety of Ministry of Education, School of Public Health, Fudan University, Shanghai 200032, China; 19111020017@fudan.edu.cn (Y.Y.); maryzaid@fudan.edu.cn (M.Z.); zhaoqi@shmu.edu.cn (Q.Z.); na.wang@fudan.edu.cn (N.W.); liuxing@fudan.edu.cn (X.L.); qiuyun2018@fudan.edu.cn (Y.Q.); zhujunjie233@163.com (J.Z.); danmoweiliangtx@gmail.com (X.T.); cuishuheng1995@outlook.com (S.C.); 2 Zhongshan Community Health Center, Shanghai 201613, China; zhongshanlijing@163.com; 3 Songjiang District Center for Disease Control and Prevention, Shanghai 201600, China; sjjkbgs@163.com (Y.J.); aries2119@163.com (Y.W.)

**Keywords:** cross-sectional study, woman health, menopause, type 2 diabetes, reproductive period

## Abstract

(1) Introduction: The available studies on the association between type 2 diabetes mellitus (T2DM) and menopause report conflicting results. (2) Objective: This study aimed to investigate the association of menopausal status, age at menopause, and length of the reproductive period with T2DM. (3) Methods: This cross-sectional study is part of the ‘China Eastern Cohort Study’, which is a community-based cohort study. Multistage, stratified, clustered sampling was used to recruit the study participants in Shanghai, China. Age at menarche and menopause was recorded, and reproductive period was calculated. Weighted logistic regression was used to calculate the prevalence ratios (PRs) with 95% confidence intervals (CIs) of T2DM. Restricted cubic splines were used to assess the relationship between age at menopause, reproductive period, and T2DM. (4) Results: A total of 20,128 women were included. The prevalence of T2DM was 13.7%. Postmenopausal women exhibited a higher prevalence of T2DM than premenopausal women (*p* < 0.001) and an unfavorable metabolic profile, including higher body mass index, hypertension, and hyperlipidemia. A higher risk of T2DM was observed in postmenopausal women (PR2.12, 95%CI: 1.79–2.51, *p* < 0.001) compared with premenopausal women, independently of confounding factors. After adjustment for confounding factors, age at menopause and reproductive period were not significantly associated with T2DM. (5) Conclusions: Postmenopausal status is associated with T2DM, while menopausal age and reproductive period are not associated with T2DM. Menopausal status should be considered during T2DM screening.

## 1. Introduction

Diabetes mellitus type 2 (T2DM) is a common endocrine disorder characterized by variable degrees of insulin resistance and deficiency, resulting in hyperglycemia [1]. T2DM is a major public health issue. The worldwide prevalence of T2DM in 2014 was 9% in men and 7.9% in women [2]. In 2021, the International Diabetes Federation [3] estimated that the number of patients with T2DM and adults with undiagnosed diabetes in China as 140.9 million and 72.8 million, respectively. Potential complications of T2DM mellitus include cardiovascular disease, neuropathy, nephropathy, retinopathy, and increased mortality [1,4], as well as changes in vasculature (macro- and microvascular complications) [5], dysfunctional immunity [6], and a chronic inflammatory state [7]. Furthermore, worldwide expenditure on diabetes-related healthcare was estimated at $760 billion in 2019 [8].

Menopause is a physiologic event characterized by the loss of ovarian activity and permanent cessation of menses, diagnosed after 12 consecutive months of amenorrhea [9,10,11]. This process occurs naturally, but can also be induced by medications, gynecologic surgery, chemotherapy, or radiation, often with sudden onset of symptoms in these circumstances [11]. The mean age of onset of natural menopause is 42–51 years [12], with perimenopausal (transitional) symptoms often seen in women aged 40–58 years. Symptoms associated with menopausal transition vary in duration and severity and may include vasomotor symptoms (hot flashes), night sweats, insomnia, vaginal atrophy, and sexual dysfunction [9,10]. Unfortunately, the menopausal transition is associated with high mortality and an increased risk of metabolic syndrome, cardiovascular disease, and osteoporosis [9,10,13,14]. Today, life expectancy is >80 years for women, meaning that a third of a woman’s life is spent in the menopausal period. Since this is a substantial amount of time, more care and attention are needed for a healthy life, free of cardiovascular disease, metabolic syndrome, and diabetes.

Recent studies reported associations between a decreasing risk of T2DM and impaired glucose metabolism with late menarche and late menopause [15,16,17,18]. On the other hand, Heianza et al. [19] reported that menopause was associated with impaired glucose metabolism, independently from age. A meta-analysis that includes six cohort studies showed that the risk of T2DM was reduced with increments in age at menopause [20], while in another prospective case-cohort study, no association was found between menopausal age and T2DM [21]. It has been reported that women with shorter reproductive periods were at higher risk of diabetes [21] and cardiovascular disease [22]. Therefore, it is still unclear whether the duration of the reproductive period is associated with T2DM, since the available studies provide conflicting results on the association of menopausal status and age with T2DM and provide little evidence on the association between reproductive period and T2DM. Moreover, few studies with large sample sizes have been conducted in China.

It is suggested that reproductive factors, such as menopausal status, age at menopause, and length of reproductive period are associated with endogenous estrogens. The protective effect of endogenous estrogens has been shown to be mainly through estrogen receptor α activation in various tissues, including the brain, the liver, skeletal muscle, adipose tissue, and pancreatic beta cells [23].

This study aimed to investigate the association of menopausal status, age at menopause, and length of the reproductive period with T2DM in a large sample of women in Shanghai, China.

## 2. Materials and Methods

### 2.1. Study Population

This cross-sectional study is part of the ‘China Eastern Cohort Study’, which is a community-based cohort study conducted by the School of Public Health of Fudan University, in collaboration with the Shanghai Songjiang Center for Disease Prevention and Control. The study protocol was approved by the ethical review committee of the School of Public Health, Fudan University (IRB approval number 2016-04-0586). Written informed consent was provided by all the recruited participants before the investigation.

The baseline evaluation was performed between June 2016 and December 2017. Multistage, stratified, clustered sampling was used to recruit the participants. Relevant information about the ‘China Eastern Cohort Study’ was previously described [24]. In the first stage, considering the population, geographic location, economic status, and participation of the region, four research areas were randomly selected, including two urban communities (Zhongshan Street and Xinqiao Town) and two rural areas (Sheshan Town and Mao Port Town). In the second stage, nine neighborhood communities and 18 neighborhood communities were randomly selected from Xinqiao Town and Zhongshan Street, and four administrative villages and 16 administrative villages were randomly selected from Sheshan Town and Maogang Town. In the third stage, all permanent residents aged 40–74 who had lived for more than 5 years in the selected neighborhood communities and administrative villages were registered.

The inclusion criteria for the present study were (1) Shanghai permanent residents aged 20–74 who had lived for more than 5 years in the designated areas, (2) female, and (3) signed the informed consent form. Among these, we excluded participants who violated the inclusion criteria (*n* = 1103); who suffered from cancer (*n* = 203); had no fasting plasma glucose (FPG) or HbA1c measurement (*n* = 171); or had missing data on physical examination, questionnaire survey, or laboratory measurements (*n* = 67). The final analysis included 20,128 participants (Supplement Figure S1).

### 2.2. Diagnostic Criteria

The diagnosis of T2DM was defined according to the International Diabetes Federation criteria [3] of FPG level ≥7.0 mmol/L, or HbA1c ≥6.5% or a previous diagnosis of T2DM. In the absence of symptoms of hyperglycemia, two abnormal tests are required for the diagnosis of diabetes mellitus. Hypertension was defined as systolic blood pressure (SBP) ≥140 mmHg and/or diastolic blood pressure (DBP) ≥90 mmHg and/or a previous diagnosis of hypertension [25]. Hyperlipidemia was defined as total cholesterol (TC) ≥6.20 mmol/L or triglycerides (TG) ≥2.30 mmol/L or high-density lipoprotein cholesterol (HDL-C) <1.00 mmol/L or low-density lipoprotein cholesterol (LDL-C) ≥4.10 mmol/L and/or a previous diagnosis of hyperlipidemia [26].

### 2.3. Definitions

The menopausal status of women was assessed using a self-reported questionnaire. Age at menopause was defined as the age at the last menstrual period, age at bilateral oophorectomy, or self-reported age at menopause. Chinese women have been reported to have an average menopausal age of 48 years [27]. Early menopause (EM) refers to menopause occurring between the age of 40 and 44. The mean age of menopause in Asian women was considered to be around 50 years old [11,15,28], and age at menopause was categorized into three groups: ≤45, 46–50, and >50 years [15]. 

The age at menarche was assessed using a self-report questionnaire and was defined as the age at the first occurrence of menstruation (in years). Age at menarche was assessed as a categorical variable (≤15, 16–18, >18 years) [17]. The reproductive period was defined as the age at menopause (years, continuous variable) minus age at menarche (years, continuous variable). The reproductive period was then assessed as a quartile categorical variable (17–31, 32–34, 35–36, and 37–47 years) [21].

Body mass index (BMI) was defined as the weight in kilograms divided by the square of height in meters (kg/m^2^). BMI was assessed as a categorical variable (≤23.9, 24–27.9, and ≥28) [29].

### 2.4. Data Collection

The date of birth, marital status, education level, smoking status, alcohol consumption, physical activity, self-reported medical history of chronic diseases, and pregnancies (either live-born or not) were assessed using a structured questionnaire by trained interviewers. Education level was defined as low (primary education or below), intermediate (secondary general or vocational education), or high (university or college). Smoking status was defined as smoking more than one cigarette per day for six months. Smoking status was classified as never, former, or current. Alcohol consumption status was recorded as yes or no. Height and weight were measured in duplicates, based on standardized protocols, and the average values were determined.

### 2.5. Statistical Analysis

All data were analyzed using Stata 16.0 (Stata Corp., College Station, TX, USA). Continuous data were converted into categories, as described above. The categorical data are presented as n (%). A Pearson chi-square test was used for the categorical data. A weighted logistic regression model was used to calculate the prevalence ratios (PRs) with 95% confidence intervals (CIs) for T2DM. The restricted cubic spline model was used to evaluate the relationship between menopausal age, reproductive period, and T2DM. Considering the large sample size, 5 knots were set on 5th, 27.5th, 50th, 72.5th and 95th percentiles of the variables (age at menopause and reproductive period) in the restricted cubic spline models, respectively [30].

## 3. Results

### 3.1. Characteristic of the Participants

A total of 20,128 participants were included in the analysis. The prevalence of T2DM was 13.7%. Participant characteristics by menopausal status and age at menopause are summarized in Table 1 and Table S1. As shown in Table 1, 5775 (28.7%) women were premenopausal. Postmenopausal women exhibited a higher prevalence of T2DM compared with premenopausal women (*p* < 0.001). Postmenopausal women were observed to have an unfavorable metabolic profile, including higher BMI, hypertension, and hyperlipidemia, compared to premenopausal women. The mean age, age at menarche, and BMI were 55.4 ± 11.1 years, 15.7 ± 1.9 years, and 24.2 ± 3.4, respectively. The mean age at menopause and reproductive period were 50.0 ± 13.9 years and 33.8 ± 4.2 years in postmenopausal women.

### 3.2. Association between Menopausal Status and T2DM

Table 2 shows the PRs for T2DM among women by menopausal status. Postmenopausal women were more likely to have T2DM (PR = 2.12, 95%CI: 1.79–2.51, *p* < 0.001) after adjusting for age and demographic factors (multivariable model 3). This study found a significant association between menopausal status and T2DM, which was independent of age, BMI, parental history of T2DM, age at menarche, pregnancies, education, smoking, alcohol, exercise, hypertension, and hyperlipidemia. Menopausal women (those with surgical or other causes) had a higher risk of T2DM (PR = 2.27, 95%CI: 1.79–2.88, *p* < 0.001) compared with naturally menopausal women (PR = 2.10, 95%CI: 1.77–2.50, *p* < 0.001) (model 3).

Table 3 shows the PRs for T2DM among women by menopausal status and age at menopause. Compared with premenopausal women, those with an age at menopause of 44–50 years had the highest PR (PR = 2.19, 95%CI: 1.83–2.61, *p* < 0.01), although postmenopausal women with a different age at menopause groups had similar PRs for T2DM.

### 3.3. Association between Age at Menopause and T2DM

**Figure 1:**
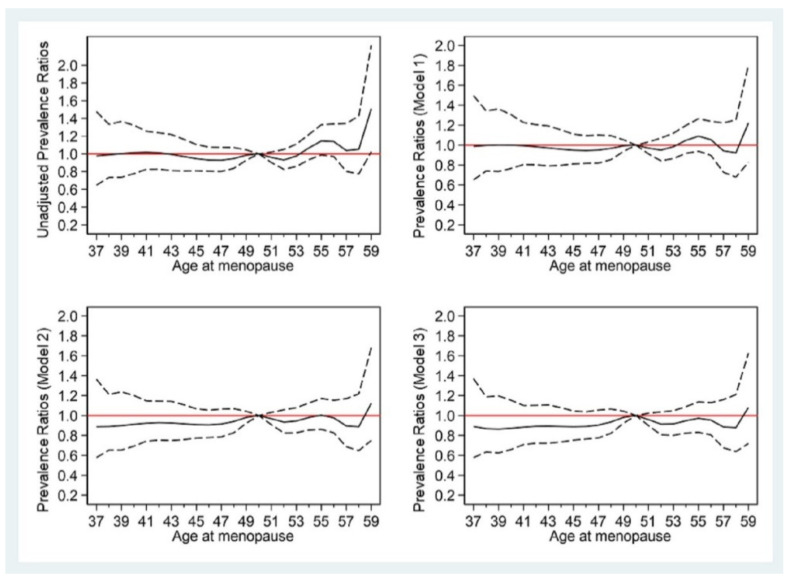
Prevalence ratios (solid line) and 95% confidence intervals (dashed lines) of age at menopause for type 2 diabetes mellitus (T2DM) (reference: 50 years old). Model 1: age; Model 2: age, BMI, parental history of T2DM, age at menarche, pregnancies, marital status; Model 3: age, BMI, parental history of T2DM, age at menarche, pregnancies, marital status, education, smoking, alcohol, exercise, hypertension, hyperlipidemia.

Table 4 and Figure 1 show the PRs for developing T2DM according to age at menopause. Compared with women with age at menopause of 46–50 years, the PRs of T2DM were not significantly different in those with menopause at ≤44 and >50 years after adjustment with models 1, 2, and 3 (Table 4). The relationship between menopausal age and T2DM risk is depicted in the restricted cubic spline curves in Figure 1.

### 3.4. Association between Reproductive Period and T2DM 

**Figure 2:**
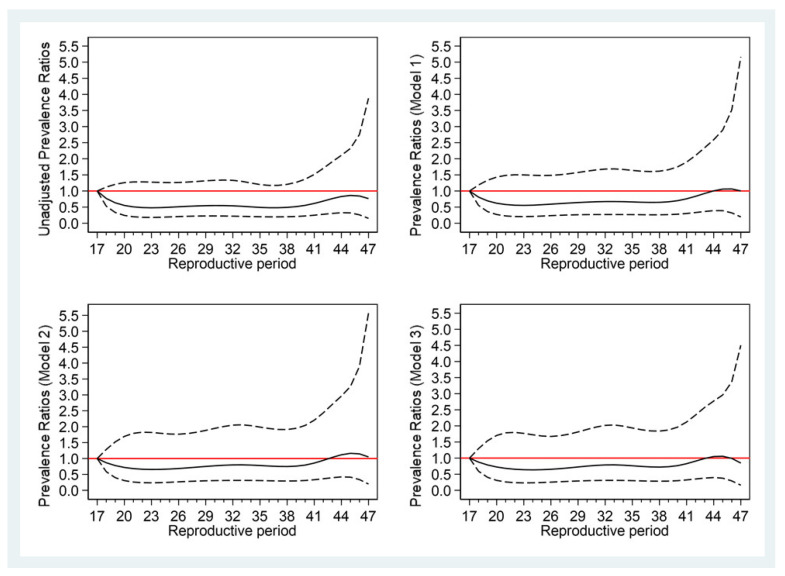
Prevalence ratios (solid line) and 95% confidence intervals (dashed lines) of the reproductive period for type 2 diabetes mellitus (T2DM) (reference: 17 years). Model 1: age; Model 2: age, BMI, parental history of T2DM, age at menarche, pregnancies, marital status; Model 3: age, BMI, parental history of T2DM, age at menarche, pregnancies, marital status, education, smoking, alcohol, exercise, hypertension, hyperlipidemia.

Table 4 and Figure 2 show the PRs for developing T2DM, according to the reproductive period. Compared with women with a reproductive period <30 years, the PRs of T2DM women were not significantly different in those with a reproductive period of 31–35 and >35 years after adjustment with models 1, 2, and 3 (Table 4). The relationship of the reproductive period with T2DM risk is depicted in the restricted cubic spline curves in Figure 2.

## 4. Discussion

The available studies on the association between T2DM and menopause report conflicting results [15,16,17,18,19,21,22]. Therefore, this study aimed to investigate the association of menopausal status, age at menopause, and length of reproductive period with T2DM in a large sample of women in Shanghai. The results from 20,165 women suggest that postmenopausal status is associated with T2DM, while menopausal age and reproductive period are not associated with T2DM. Menopausal status should be considered during T2DM screening.

Whether menopausal status is associated with T2DM independently of age and other confounding factors remains controversial [16,17,18]. A Dutch study found that postmenopausal women had a higher risk of T2DM, even after adjustments for important covariates [31]. In a cross-sectional study in Jiangsu, China, postmenopausal women had a lower risk of high FPG and HbA1c than premenopausal women [17]. These studies suggest that postmenopausal status is associated with T2DM, as observed in the present study. On the other hand, a study in Vietnam showed that the association between menopausal status and T2DM was no longer significant after adjustment for age, occupation, BMI, physical activity, parental history of T2DM, residential status, and hypertension [16]. The conflicting results among studies are likely due to differences in model adjustment, sample population, and/or genetic features among women in different countries.

In this study, no significant association was found between age at menopause and T2DM, after adjusting for several potential confounders (models 1, 2, and 3). Previous research into the relationship between menopausal age and T2DM yielded inconsistent and contradictory results. One study found that compared with women who had their menopause at 45–52 years, women with menopause after 53 years had a 21% higher risk of T2DM [32], but no significant association was found between advanced menopausal age and T2DM when adjusting for hypertension. In another Chinese cohort study, for every 1-year delay in the age at menopause, the incidence of T2DM decreased by 2% (0.98 (0.97–0.99)), when adjustment was made for age, BMI, and several potential confounding factors [18]. In that study, the OR for T2DM was 1.20 (1.03–1.39) for women whose age at menopause was 46–52 years [18]. As reviewed by Brand et al. [21], younger age at menopause could be associated with a lower risk of T2DM. A prospective case-cohort study in Europe found that after multivariable adjustment, women whose menopausal age was <40 had a higher risk of T2DM, and the hazard ratios (HRs) (95%CI) were 1.32 (1.04–1.69), 1.09 (0.90–1.31), 0.97 (0.86–1.10), and 0.85 (0.70–1.03), respectively [33]. In this study, the association between the length of the reproductive period and T2DM was not significant. Only one previous study reported an association between the reproductive period and T2DM [21]. After adjustment for age, BMI, smoking, alcohol, physical activity, education, and pregnancies, women with a shorter reproductive period had a higher risk of developing T2DM, and the HR for SD decrease in the reproductive period was 1.06 (1.01–1.02) [21]. This study can supplement the diversity of results on this issue, and this is the first study in China to discuss the relationship between reproductive period and T2DM. Again, it is possible that the association between T2DM and age at menopause varies among countries and could be influenced by various factors, including diet and lifestyle habits. Additional studies are necessary to address this issue comprehensively.

Day et al. [34] suggested that menopause might be associated with T2DM and DNA damage repair. Therefore, menopause could be a marker of aging of the somatic (non-reproductive) tissues. Future studies should consider examining genetic factors related to T2DM. One possible impact of menopause on T2DM may relate to the characteristics of the menopausal transition, which vary among women [35]. As women age, their risk of T2DM increases, and as they reach menopausal age, a drop in estrogen combined with other risk factors such as obesity predisposes them to a greater risk for T2DM. Changes in body fat distribution before and after menopause might reduce tissue insulin sensitivity and glucose tolerance, which increase T2DM risk [36]. Postmenopausal women with hormone replacement therapy were reported to have a low risk of developing cardiovascular morbidity and mortality [37]. Animal studies have found that hormone replacement therapy can decrease the postmenopausal growth of fat mass by about 60% [38]. However, it is reported that in mainland China the rate of regularly using hormone replacement therapies (for 1 year and above) was 1.1%. Therefore, hormone replacement therapies should have had a small impact on the results of the present study. Premenopausal women affected by ovarian cancer experience acute surgical- or chemotherapy-induced menopause, leading to more prominent menopausal symptoms [39]. Moreover, naturally menopausal women are reported to possess health advantages over artificially-induced menopausal women, which might suggest that senescent ovaries provide health advantages [40]. In menopause, social and environmental factors such as lower education, smoking habit, and unemployment can affect the menopausal age, affecting the association of age at menopause and T2DM. Regular physical activity can prevent weight gain and adverse changes in body composition and body fat distribution in menopausal women. Studies have shown that higher levels of recreational physical activity, including walking, are associated with a significant reduction of heart failure risk in community-dwelling aged women [41,42]. Future studies should focus on the effect of interventions, such as regular physical activity and diet, on metabolic health problems such as T2DM, obesity, and hypertension. 

This study has several strengths. First, it used a community-based sample population, allowing the results to be generalizable to other groups. The sample size was large, and the background of the population was diverse. This study has a wide age range of population, with both premenopausal and postmenopausal women. Strict quality control systems were implemented by recording the process of the questionnaire and double-checking [43]. The diagnostic criteria for T2DM used were consistent with international standards. Furthermore, this is the first study in China to investigate the relationship between the reproductive period and T2DM.

However, this study has several limitations. The criteria for T2DM diagnosis used did not include the data of oral glucose tolerance test (OGTT), because of the difficult logistics of this test in the context of large-scale epidemiological studies, which may have resulted in the underestimation of the association between menopausal status and T2DM. Further studies using the updated T2DM criteria that incorporates OGTT measurements would strengthen our findings. Using only single measurements of HbA1c and FPG in the cross-sectional analyses is, indeed, another limitation of our study. Further evaluations, more accurately discerning T2DM, are necessary. The data on menopause were collected by self-reported questionnaire; therefore, there is a possibility of information bias. This study is a cross-sectional survey; therefore, the ability to elucidate the causal inference between menopausal status, age at menopause, reproductive period, and T2DM was limited. Prospective cohort studies are needed to validate the results. Hormone therapy was not included in the adjustment model, yet, it was associated with a lower incidence of T2DM in postmenopausal women [44] and improvements in pathway-selective insulin resistance in surgically menopausal women [45]. Nevertheless, in Chinese menopausal women, the prevalence of hormone replacement therapy was only 2.1% [46]. Furthermore, this study did not consider menopausal women (surgical or other cause) separately from naturally menopausal women. Future studies comparing these two groups of menopausal women are warranted.

## 5. Conclusions

In this community-based study of Chinese women, the menopausal status in women was associated with T2DM after adjustment for age, BMI, parental history of T2DM, pregnancies, marital status, history of T2DM, education level, age at menarche, hypertension, hyperlipidemia, alcohol, smoking, and physical activity. There were no associations between T2DM and age at menopause or the reproductive period. Due to the association between menopause and T2DM, screening for T2DM in menopausal women should be considered. Our findings indicated that postmenopausal women have a higher risk of diabetes. Early surveillance of diabetes in postmenopausal women is needed, so that undiagnosed women are diagnosed earlier and protected from diabetic complications.

## Figures and Tables

**Table 1:** Characteristics of the women with menopausal status (*n* = 20,128).

Characteristics	Menopausal Status				*p*
	Pre-Menopause	Post-Menopause Age			
	(*n* = 5775)	≤44 (*n* = 1682)	45–50 (*n* = 6554)	>50 (*n* = 6117)	
Age					<0.001
≤55	5650 (97.8%)	446 (26.5%)	1916 (29.2%)	1403 (22.9%)	
56–60	101 (1.7%)	351 (20.9%)	1389 (21.2%)	1561 (25.5%)	
61–65	24 (0.4%)	368 (21.9%)	1519 (23.2%)	1602 (26.2%)	
>65	0	517 (30.7%)	1730 (26.4%)	1551 (25.4%)	
Marital status					<0.001
Married	5423 (93.9%)	1496 (88.9%)	5980 (91.2%)	5542 (90.6%)	
Unmarried/Divorced/Widowed	352 (6.1%)	186 (11.1%)	574 (8.8%)	575 (9.4%)	
Pregnancies (times)					<0.001
0	253 (4.4%)	14 (0.8%)	38 (0.6%)	16 (0.3%)	
1	1704 (29.5%)	226 (13.4%)	1088 (16.6%)	944 (15.4%)	
2	2085 (36.1%)	665 (39.5%)	2610 (39.8%)	2569 (42.0%)	
≥3	1733 (30.0%)	777 (46.2%)	2818 (43.0%)	2588 (42.3%)	
Age at menarche (years)					<0.001
≤15	3980 (68.9%)	602 (35.8%)	2470 (37.7%)	2014 (32.9%)	
16–18	1709 (29.6%)	837 (49.8%)	3222 (49.2%)	3330 (54.4%)	
>18	86 (1.5%)	243 (14.4%)	862 (13.2%)	773 (12.6%)	
Reproductive period (years)					<0.001
17–31	N/A	1697 (97.0%)	1968 (29.0%)	53 (0.8%)	
32–34	N/A	53 (3.0%)	2890 (42.5%)	776 (12.3%)	
35–36	N/A	0	1720 (25.3%)	1996 (31.5%)	
37–47	N/A	0	215 (3.2%)	3504 (55.4%)	
Education					<0.001
Primary or below	932 (16.1%)	1184 (70.4%)	4209 (64.2%)	3949 (64.6%)	
Secondary or vocational	3606 (62.4%)	489 (29.1%)	2304 (35.2%)	2144 (35.0%)	
University or college	1237 (21.4%)	9 (0.5%)	41 (0.6%)	24 (0.4%)	
Body mass index (kg/m^2^)					<0.001
<24	3743 (64.8%)	755 (44.9%)	3111 (47.5%)	2721 (44.5%)	
24–27.9	1515 (26.2%)	679 (40.4%)	2549 (38.9%)	2502 (40.9%)	
≥28	517 (9.0%)	248 (14.7%)	894 (13.6%)	894 (14.6%)	
Smoking					0.063
Never	5750 (99.6%)	1678 (99.8%)	6541 (99.8%)	6106 (99.8%)	
Former	6 (0.1%)	2 (0.1%)	1 (<1%)	3 (<1%)	
Current	19 (0.3%)	2 (0.1%)	12 (0.2%)	8 (0.1%)	
Alcohol					0.063
No	5719 (99.0%)	1671 (99.3%)	6516 (99.4%)	6077 (99.3%)	
Yes	56 (1.0%)	11 (0.7%)	38 (0.6%)	40 (0.7%)	
Exercise					0.019
No	3854 (66.7%)	1156 (68.7%)	4546 (69.4%)	4170 (68.2%)	
Yes	1921 (33.3%)	526 (31.3%)	2008 (30.6%)	1947 (31.8%)	
T2DM	231 (4.0%)	288 (17.1%)	1140 (17.4%)	1093 (17.9%)	<0.001
Family history of T2DM	765 (13.2%)	229 (13.6%)	775 (11.8%)	786 (12.8%)	0.058
Hypertension	1395 (24.2%)	1004 (59.7%)	3755 (57.3%)	3766 (61.6%)	<0.001
Hyperlipidemia	793 (13.7%)	515 (30.6%)	1855 (28.3%)	1791 (29.3%)	<0.001
Menopause					<0.001
Natural	—	1258 (74.8%)	6217 (94.9%)	5988 (97.9%)	
Surgical	—	424 (25.2%)	337 (5.1%)	129 (2.1%)	

The data are presented as n (%). T2DM: type 2 diabetes mellitus. *p*-values for differences between groups were obtained from Chi-square tests of frequencies in the respective characteristic among menopausal status.

**Table 2:** Prevalence ratios (PR) (95%CI) for T2DM in premenopausal and postmenopausal women.

Characteristics	Model 1		Model 2		Model 3	
	PR (95% CI)	*p*	PR (95% CI)	*p*	PR (95% CI)	*p*
Premenopausal women (*n* = 5775)	1	-	1	-	1	-
Postmenopausal women (all) (*n* = 14,390)	3.12 (2.65,3.67)	<0.001	2.83 (2.39,3.34)	<0.001	2.12 (1.79,2.51)	<0.001
Postmenopausal women (natural) (*n* = 13,463)	3.07 (2.60,3.61)	<0.001	2.81 (2.37,3.33)	<0.001	2.10 (1.77,2.50)	<0.001
Postmenopausal women (surgical or other cause) (*n* = 890)	3.64 (2.90,4.57)	<0.001	3.00 (2.37,3.79)	<0.001	2.27 (1.79,2.88)	<0.001

Model 1: age; Model 2: age, BMI, parental history of T2DM, age at menarche, pregnancies, marital status; Model 3: age, BMI, parental history of T2DM, age at menarche, pregnancies, marital status, education, smoking, alcohol, exercise, hypertension, hyperlipidemia. PR: prevalence ratio; CI: confidence interval; BMI: body mass index; T2DM: type 2 diabetes mellitus.

**Table 3:** Prevalence ratios (PR) (95%CI) for T2DM in premenopausal and postmenopausal women with different age at menopause.

Characteristics	Model 1		Model 2		Model 3	
	PR (95% CI)	*p*	PR (95% CI)	*p*	PR (95% CI)	*p*
Premenopausal women	1	-	1	-	1	-
Age at menopause (postmenopausal women)						
≤44 years	3.00 (2.45,3.68)	<0.001	2.64 (2.14,3.25)	<0.001	1.94 (1.57,2.40)	<0.001
45–50 years	3.13 (2.64,3.71)	<0.001	2.89 (2.43,3.44)	<0.001	2.19 (1.83,2.61)	<0.001
>50 years	3.15 (2.65,3.74)	<0.001	2.81 (2.35,3.35)	<0.001	2.09 (1.74,2.50)	<0.001

Model 1: age; Model 2: age, BMI, parental history of T2DM, age at menarche, pregnancies, marital status; Model 3: age, BMI, parental history of T2DM, age at menarche, pregnancies, marital status, education, smoking, alcohol, exercise, hypertension, hyperlipidemia. PR: prevalence ratio; CI: confidence interval; BMI: body mass index; T2DM: type 2 diabetes mellitus.

**Table 4:** Prevalence ratios (PR) (95% CI) for T2DM in postmenopausal women with different age at menopause and reproductive period.

Characteristics	Model 1		Model 2		Model 3	
	PR (95% CI)	*p*	PR (95% CI)	*p*	PR (95% CI)	*p*
Age at menopause						
≤44	0.96 (0.83,1.11)	0.572	0.91 (0.79,1.06)	0.230	0.89 (0.77,1.03)	0.126
45–50	1	.	1	.	1	.
>50	1.01 (0.92,1.10)	0.891	0.98 (0.89,1.07)	0.624	0.96 (0.87,1.06)	0.428
Per 1-year increase	1.01 (1.00,1.02)	0.184	1.00 (0.99,1.01)	0.555	1.00 (0.99,1.01)	0.618
Reproductive period (years)						
17–31	1	.	1	.	1	.
32–34	1.09 (0.97,1.24)	0.154	1.11 (0.98,1.26)	0.106	1.11 (0.98,1.27)	0.101
35–36	0.97 (0.88,1.06)	0.621	0.97 (0.88,1.08)	0.665	0.97 (0.88,1.08)	0.712
37–47	1.06 (0.94,1.20)	0.356	1.03 (0.90,1.18)	0.678	1.02 (0.89,1.17)	0.772
Per 1-year increase	1.01 (1.00,1.02)	0.284	1.00 (0.99,1.01)	0.534	1.00 (0.99,1.01)	0.594

Model 1: age; Model 2: age, BMI, parental history of T2DM, age at menarche, pregnancies, marital status; Model 3: age, BMI, parental history of T2DM, age at menarche, pregnancies, marital status, education, smoking, alcohol, exercise, hypertension, hyperlipidemia. PR: prevalence ratio; CI: confidence interval; BMI: body mass index; T2DM: type 2 diabetes mellitus.

## Data Availability

All data generated or analyzed during this study are available from the corresponding author upon reasonable request.

## Supplementary Materials

The following supporting information can be downloaded at: https://www.mdpi.com/article/10.3390/ijerph19021019/s1, Figure S1: Flowchart of the study population; Table S1: Characteristics of the women among menopausal status (*n* = 20,128).
