# Supplementary material for: Association between Reproductive Factors and Type 2 Diabetes: A Cross-Sectional Study
Source: Int J Environ Res Public Health. 2022 Jan 17;19(2):1019. doi: 10.3390/ijerph19021019 (PMC8775663; doi:10.3390/ijerph19021019)
Supplement: Supplementary file 1 [file ijerph-19-01019-s001.zip › ijerph-1529105-supplementary.pdf]

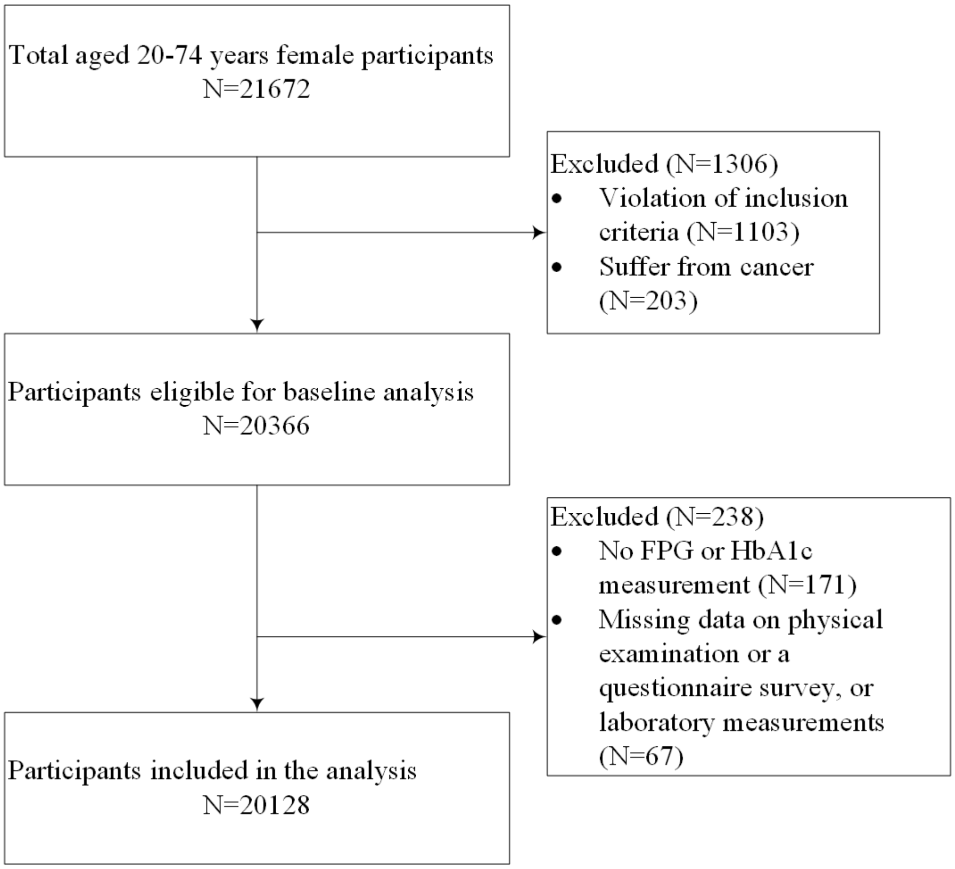

Figure S1. Flowchart of the study population

Table S1. Characteristics of the women among menopausal status (n = 20,128)

	Menopausal status			
**Characteristics**	**Pre-menopause**	**Postmenopausal (natural)**	**Postmenopausal (surgical or other cause)**	***P***
	**(*n* = 5775)**	**(*n* = 13,463)**	**(*n* = 890)**	
Age				<0.001
≤55	5650 (97.8%)	3388 (25.2%)	377 (42.4%)	
56-60	101 (1.7%)	3062 (22.7%)	239 (26.9%)	
61-65	24 (0.4%)	3321 (24.7%)	168 (18.9%)	
>65	0 (0.0%)	3692 (27.4%)	106 (11.9%)	
Marital status				<0.001
Married	5423 (93.9%)	12195 (90.6%)	823 (92.5%)	
Unmarried/Divorced/Widowed	352 (6.1%)	1268 (9.4%)	67 (7.5%)	
Pregnancies (times)				<0.001
0	253 (4.4%)	64 (0.5%)	4 (0.4%)	
1	1704 (29.5%)	2097 (15.6%)	161 (18.1%)	
2	2085 (36.1%)	5495 (40.8%)	349 (39.2%)	
≥3	1733 (30.0%)	5807 (43.1%)	376 (42.2%)	
Age at menarche (years)				<0.001
≤15	3980 (68.9%)	4715 (35.0%)	371 (41.7%)	

16-18	1709 (29.6%)	6946 (51.6%)	443 (49.8%)	
>18	86 (1.5%)	1802 (13.4%)	76 (8.5%)	
Reproductive period (years)				<0.001
17-31	NA	3030 (22.5%)	542 (60.9%)	
32-34	NA	3413 (25.4%)	168 (18.9%)	
35-36	NA	3487 (25.9%)	113 (12.7%)	
37-47	NA	3533 (26.2%)	67 (7.5%)	
Education				<0.001
Primary or below	932 (16.1%)	8835 (65.6%)	507 (57.0%)	
Secondary or vocational	3606 (62.4%)	4564 (33.9%)	373 (41.9%)	
University or college	1237 (21.4%)	64 (0.5%)	10 (1.1%)	
Body mass index (kg/m^2)				<0.001
<24	3743 (64.8%)	6203 (46.1%)	384 (43.1%)	
24-27.9	1515 (26.2%)	5357 (39.8%)	373 (41.9%)	
≥28	517 (9.0%)	1903 (14.1%)	133 (14.9%)	
Smoking				0.044
Never	5750 (99.6%)	13437 (99.8%)	888 (99.8%)	
Former	6 (0.1%)	5 (<1%)	1 (0.1%)	
Current	19 (0.3%)	21 (0.2%)	1 (0.1%)	
Alcohol				0.016
No	5719 (99.0%)	13377 (99.4%)	887 (99.7%)	
Yes	56 (1.0%)	86 (0.6%)	3 (0.3%)	
Exercise				0.020
No	3854 (66.7%)	9260 (68.8%)	612 (68.8%)	
Yes	1921 (33.3%)	4203 (31.2%)	278 (31.2%)	
T2DM	231 (4.0%)	2361 (17.5%)	160 (18.0%)	<0.001
Family history of T2DM	765 (13.2%)	1628 (12.1%)	162 (18.2%)	<0.001
Hypertension	1395 (24.2%)	8030 (59.6%)	495 (55.6%)	<0.001
Hyperlipidemia	793 (13.7%)	3908 (29.0%)	253 (28.4%)	<0.001

The data are presented as n (%). T2DM: type 2 diabetes mellitus. P-vaues for differences between groups were obtained from Chi-square tests of frequencies in the respective characteristic among menopausal status.
